# Health inequalities in access to keloid scar surgery: An analysis of funding criteria and their impact across England

**DOI:** 10.1016/j.jpra.2025.04.014

**Published:** 2025-05-06

**Authors:** Hermione Richardson, Sunima Basnet, Deniz Rad, Roshan Vijayan

**Affiliations:** a University of Cambridge, School of Clinical Medicine, United Kingdom; b East & North Hertfordshire NHS Trust, Lister Hospital, Coreys Mill Lane, Stevenage SG1 4AB, United Kingdom

**Keywords:** Keloid, Funding, Criteria, Healthcare

## Abstract

**Background:**

Keloid scars, which are significantly more common in ethnic minorities, are challenging to treat in England because of funding policies. Overly restrictive funding criteria can have a disproportionate adverse effect on those most prone to keloid, resulting in health inequality.

**Methods:**

This cross-sectional study investigated the regional variations in funding criteria for keloid scar surgery across the 42 integrated care boards (ICBs) of England and, through Freedom of Information requests, the number of corresponding applications and approvals in 2023. Potential associations with the Black, Asian and minority ethnic composition of ICB populations were explored.

**Results:**

Funding criteria were highly restrictive across all ICBs. Keloid scar surgery applications were almost universally low, with some ICBs reporting <5 or no applications. Acceptance rates were generally low. There was no significant overall correlation between the ethnic proportion of ICBs’ populations and number of applications or approval rates.

**Conclusions:**

Our findings confirmed that access to keloid surgery is highly restricted nationwide, a situation that likely disproportionately disadvantages the ethnic minorities. The near total lack of ethnicity data collection by ICBs obstructs the full characterisation of this issue. We propose exempting keloids from funding restrictions or at least revising the criteria to account for their symptomatic and psychosocial impact, to help achieve more equitable healthcare policies.

## Introduction

Access to 58 procedures and investigations is currently restricted by the national evidence-based intervention (EBI) policy, with further procedures under the regional jurisdictions of integrated care boards (ICBs), subject to varying criteria. The stated aim of funding criteria is to reduce expenditure which implicitly results in restricted access.

Several keloids are not linear raised scars but bulky masses, often conspicuously situated, which can cause embarrassment, social withdrawal and symptoms of discomfort and pruritis. Unlike other soft tissue masses such as lipomas and epidermoid cysts, which are covered under national EBI and operable if they satisfy one of the 10 listed criteria,^1^ keloids are often covered by regional policies determined by ICBs in the UK. Non-operative management with intralesional steroid may be appropriate in small keloids, but may not be sufficiently effective in large keloids, while inflicting multiple painful episodes. By contrast, surgical debulking has favourable efficacy and recurrence rates with adjunctive steroid.^2^

Health inequalities, as defined by the National Institute for Health and Care Excellence, are systematic, unfair and avoidable differences in health outcomes between different groups in society. Counterintuitively, treating everyone the same does not necessarily result in equality or fairness. Natural variations within a population can indicate that equal treatment leads to inequity. An example is keloid scars, where stringent funding criteria over recent years has impeded the access to surgery. The criteria apply equally to everyone, yet keloids are estimated to have a 15 times higher incidence in Black individuals compared to Caucasians.^3^ Additionally, evidence indicates that scar-related psychosocial sequelae and burden of symptoms are higher in the Black population.^4^

This analysis explored the regional variation across England in funding criteria for keloid scar surgery, and the corresponding number of applications and approvals by ICBs in 2023. We collected ethnic demographic data to explore any association, with the hypothesis that ICBs with higher Black, Asian and minority ethnic (BAME) populations would receive more applications, and that in a fair system, the approval rates across ICBs would be comparable.

## Materials and Methods

The study employed a cross-sectional design involving all 42 ICBs across England. Between January and March 2024, Freedom of Information (FOI) requests were submitted by email to each ICB.

Requests outlined the purpose of the study and were accompanied by a standardised survey. The survey comprised 13 questions to gather information regarding the number of applications and approvals for keloid scar surgery in the year 2023, their anatomical locations, the reason for rejections, and the ethnicity or Fitzpatrick skin types of all applications (Appendix A).

Follow-up emails were sent and requests for alternative survey formats were accommodated.

### Development of Restrictiveness Scale

The funding criteria for each of the 42 ICBs across England regarding keloid scar surgery were accessed via their websites and FOI requests. Criteria were collated and tabulated.

A scale from 1 to 4 was developed to reflect the degree of restrictiveness in descending order, with 1 being the most restrictive and 4 the least. The most common features of keloid scar presentations in the literature, such as adverse cosmesis, discomfort and pruritis,^5^^,^^6^^,^^7^ were determined to constitute a relatively lenient criterion, while uncommon or rare features constituted stricter criteria. Some criteria in use—bleeding, obstruction of orifices, limitation in mobility, infection and malignancy—seldom, if at all used, were featured in the literature.

An ICB’s overall level of restriction is only as restrictive as the least restrictive non-conditional constituent; thus, if facial keloids are permissible under category 3, the other accompanying non-conditional criteria, though more restrictive, are somewhat mitigated.

1. **Individual Funding Request (IFR) only:** Keloid scar treatments are not routinely funded, so an IFR must be submitted with the evidence of ‘clinical exceptionality’
2. **Prior approval process outlining functional disablement or disfigurement:** Funding requires significant functional symptoms such as obstructing an orifice, limiting mobility or is large enough to be considered disfiguring.
3. **Prior approval process for rare presentations:** Applications are accepted if the keloid scar is limited to the face or presents with bleeding, severe pain, infection, malignancy and/or is not caused by piercing.
4. **Prior approval process other than rare presentations:** Treatment funding is provided for keloid scars which present with symptoms such as pruritus or discomfort, other than those listed above which are more common presentations.

### Data analysis

#### Ethnicity data

A custom dataset was obtained from the Office for National Statistics (ONS) website, comprising ethnicity data for individuals within each ICB, derived from the 2021 census. This enabled the calculation of the ethnic breakdown within each ICB including the percentage of individuals identifying as BAME.

#### Handling of the FOI data

Among the 42 FOI requests submitted, 40 responses were received. Among the 40 responses, 24 answered at least questions 1-5 and 8. Others declined, in most cases citing unavailability of data or patient confidentiality concerns due to low numbers of applications. Question 11 was refused in all but 4 cases as the ICB did not hold this information, and questions 12 and 13 were refused in all but 7 cases as hypotheticals are excluded in the FOI guidelines.

ICBs with ≤5 applications were omitted from the calculation of acceptance rates for keloid scar treatment funding, as such small numbers were deemed insufficient to provide a representative percentage. This left 14 ICBs with sufficient data to calculate the acceptance rates.

In some cases, the ICB provided a range (i.e. <5 or <10) to maintain patient confidentiality when working with low values. Therefore, ‘<5’ was interpreted as representing a value between 1 and 4 (as ICBs reported values of 0 where applicable), and ‘<10’ as between 5 and 9. These estimates were then used to calculate a range for approval rates and applications per million people. Population figures were based on the 2021 census.

## Results

Analysis of individual ICB funding criteria for keloid scar surgery reveals that it is not freely available anywhere in the country. Variable funding criteria apply, with 22 of the 40 ICBs having the highest category of restriction on our scale, with keloids not being considered without an IFR. Only 4 out of 40 ICBs that responded, recorded ethnicity or Fitzpatrick skin type data of the applicants.

**Figure 1:**
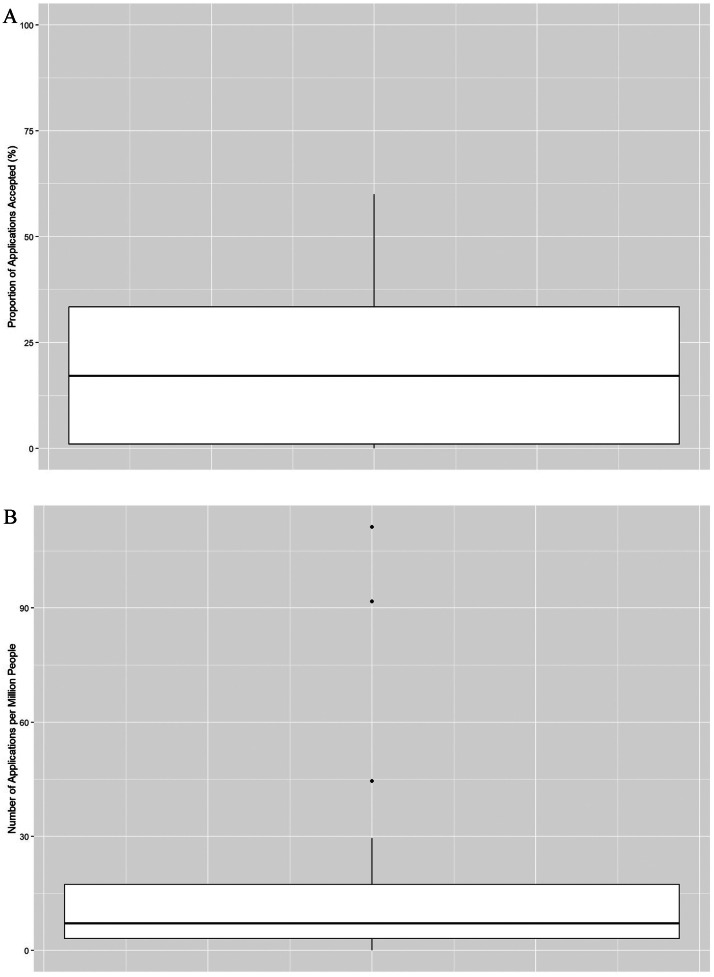
A. Box plot showing the distribution of approval rates across ICBs B. Box plot showing the distribution of the number of applications per million people across ICBs.

Applications for keloid surgery across the country demonstrated large variations. The mean number of applications per million people was 17.5 (range = 0 to 111, SD = 28.2; Figure 1A). The mean approval rate was 21.4% (range = 0 to 58.8%, SD = 20.6%; Figure 1B).

### Ethnicity of ICB population and corresponding restrictiveness of funding criteria

**Figure 2:**
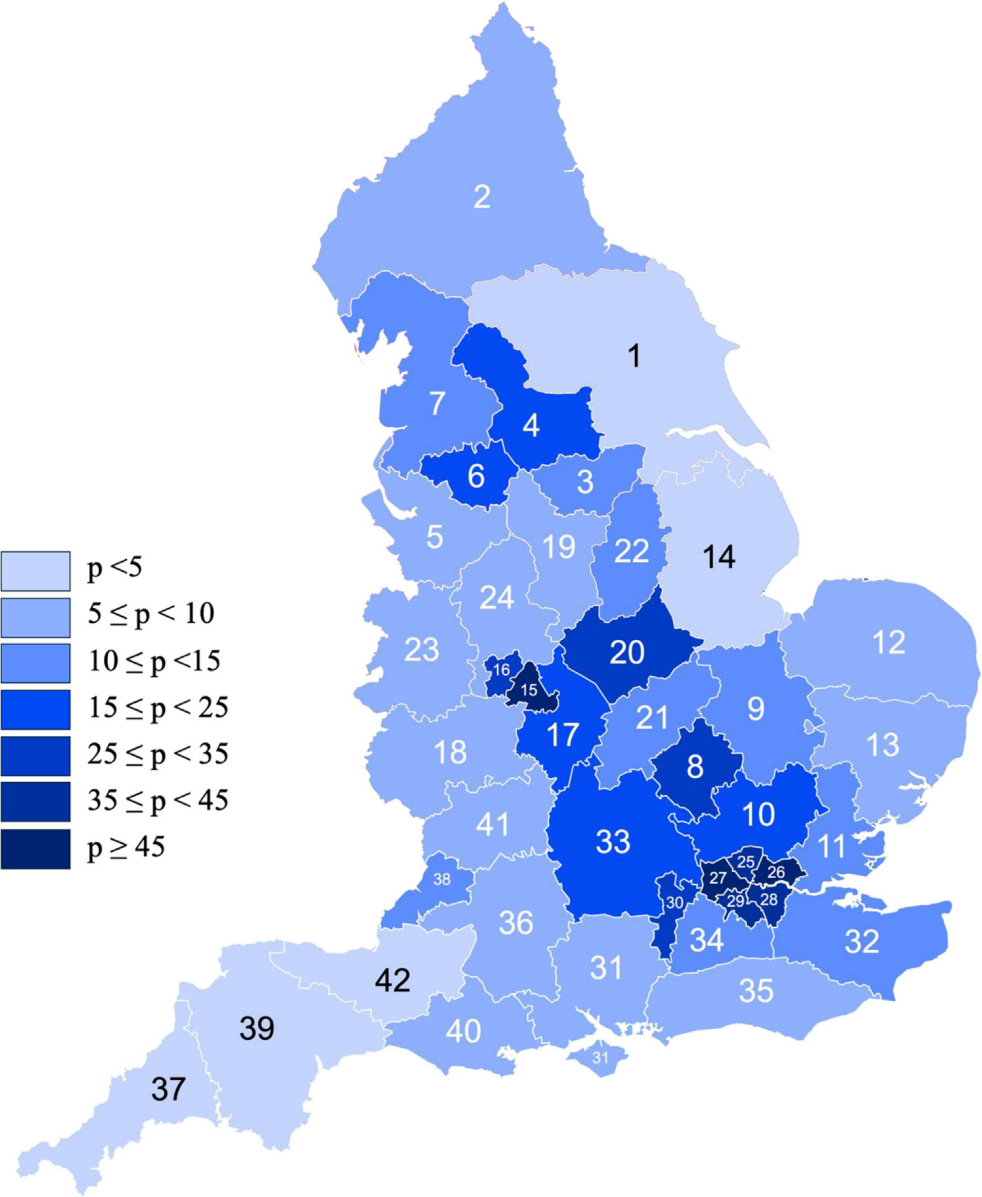
Choropleth map showing the percentage of BAME population, p, in each ICB.

**Figure 3:**
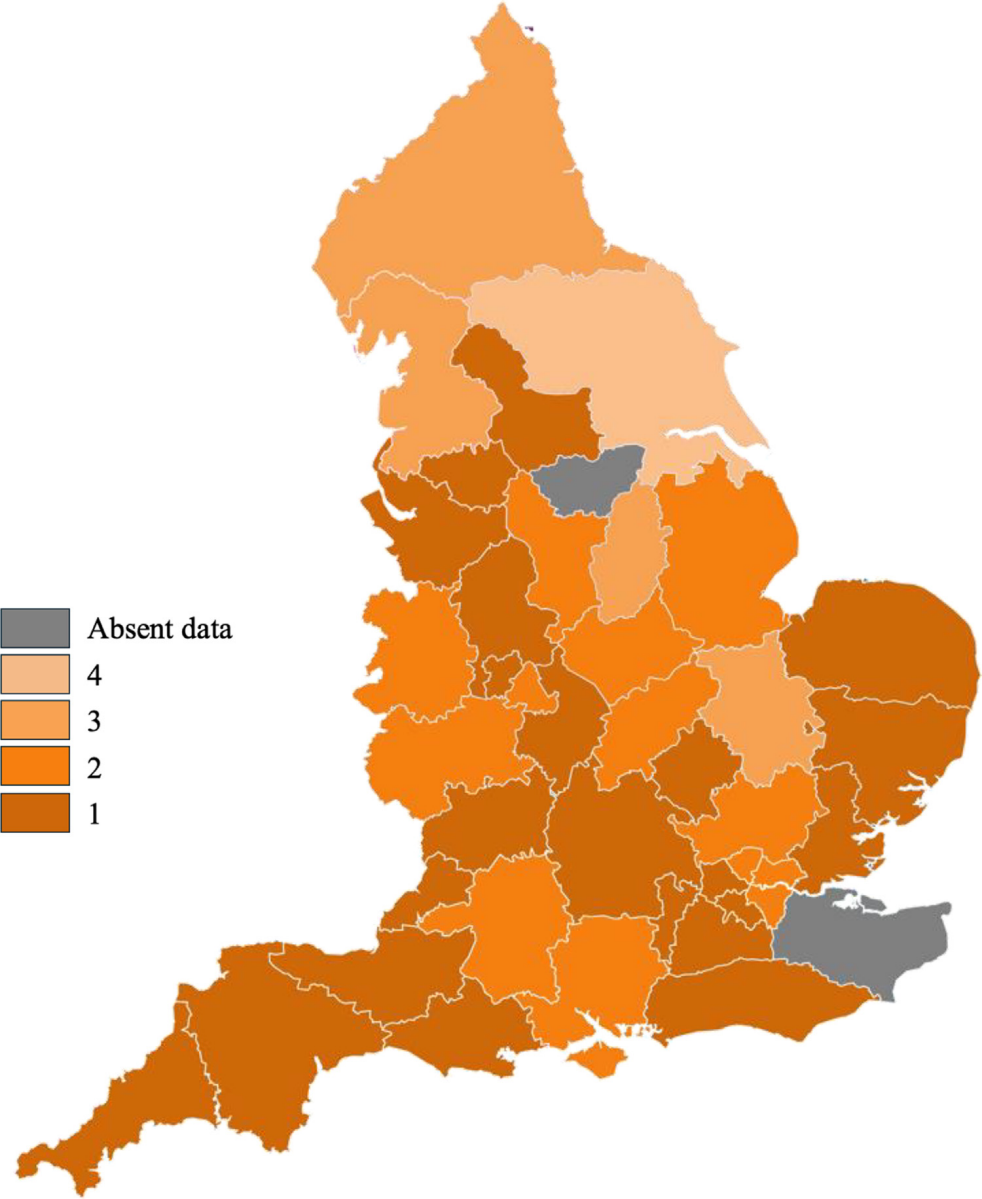
Choropleth map showing the extent of restrictive keloid scar removal funding criteria are in each ICB, rated on scale of 1-4 where 1= IFR only, 2= prior approval process outlining functional disablement or disfigurement, 3= prior approval process for rare presentations and 4= prior approval process other than rare presentations.

**Figure 4:**
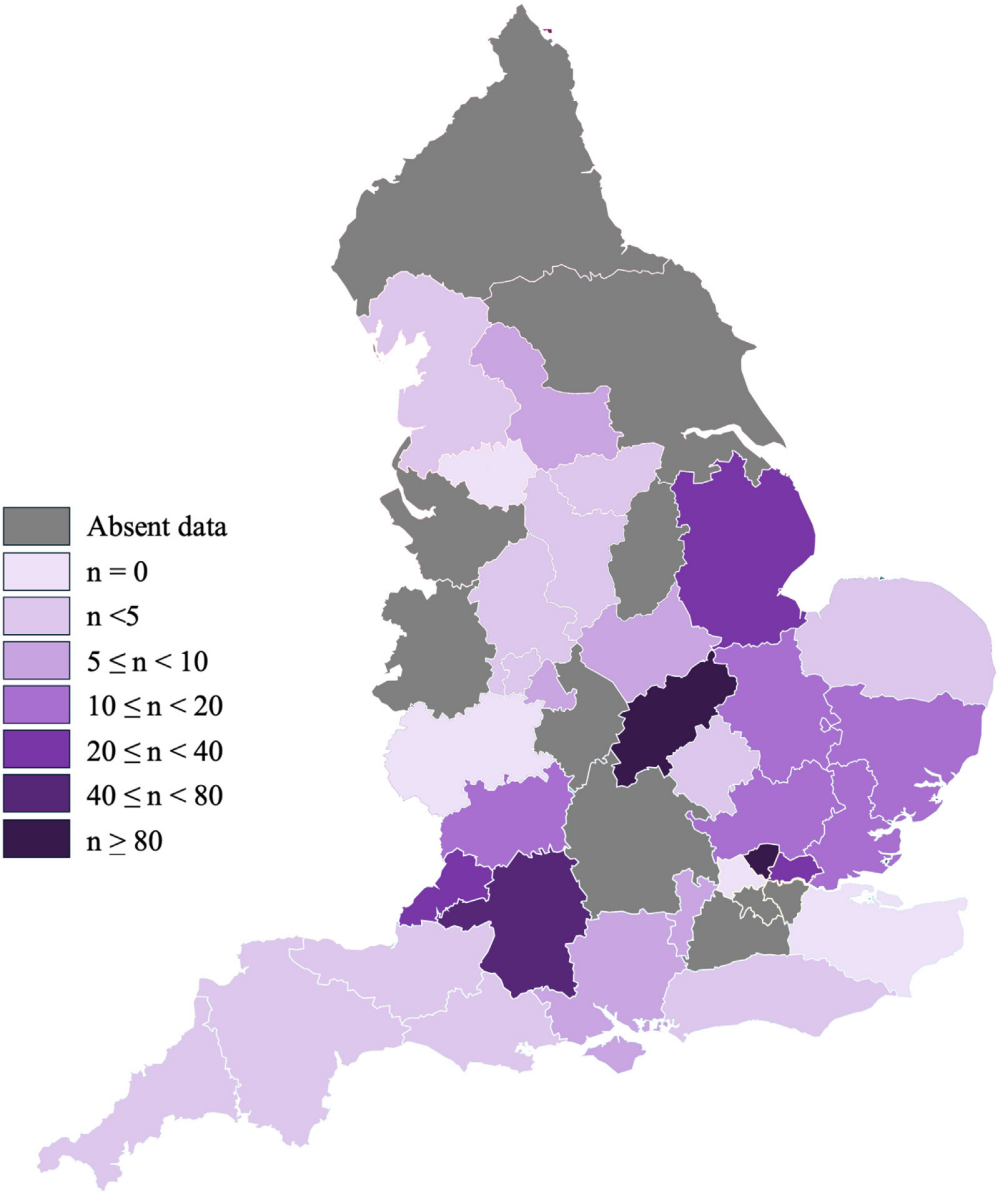
Choropleth map showing the number of keloid scar removal funding applications per million people, n, in each ICB.

Choropleth maps illustrating the distribution of the BAME population across the ICBs (Figure 2) were compared with the restrictiveness of the keloid scar surgery funding criteria (Figure 3). The criteria were generally restrictive regardless of the variations in the BAME population. NHS Lincolnshire (14), with a BAME population percentage <5%, had criteria as restrictive as that of NHS Leicester, Leicestershire and Worcestershire (20), where the BAME population proportion ranged from 35% to 45%. An additional choropleth map (Figure 4) illustrates the number of keloid surgery applications per million people. No significant correlation between the number of applications and strictness of funding criteria was observed with the limited data.

**1=** Humber and North Yorkshire, **2=** North East and North Cumbria, **3=** South Yorkshire, **4=** West Yorkshire, **5=** Cheshire and Merseyside, **6=** Greater Manchester, **7=** Lancashire and South Cumbria, **8=** Bedfordshire, Luton and Milton Keynes, **9=** Cambridgeshire and Peterborough, **10=** Hertfordshire and West Essex, **11=** Mid and South Essex, **12=** Norfolk and Waveney, **13=** Suffolk and North East Essex, **14=** Lincolnshire, **15=** Birmingham and Solihull, **16=** Black Country, **17=** Coventry and Warwickshire, **18=** Herefordshire and Worcestershire, **19=** Derbyshire, **20=** Leicester, Leicestershire and Rutland, **21=** Northamptonshire, **22=** Nottingham and Nottinghamshire, **23=** Shropshire, Telford and Wrekin, **24=** Staffordshire and Stoke-on-Trent, **25=** North Central London, **26=** North East London, **27=** North West London, **28=** South East London, **29=** South West London, **30=** Frimley, **31=** Hampshire and Isle of Wight, **32=** Kent and Medway, **33=** Buckinghamshire, Oxfordshire and Berkshire West, **34=** Surrey Heartlands, **35=** Sussex, **36=** Bath and North East Somerset, Swindon and Wiltshire, **37=** Cornwall and the Isles of Scilly, **38=** Bristol, North Somerset and South Gloucestershire, **39=** Devon, **40=** Dorset, **41=** Gloucestershire and **42=** Somerset.

### Applications in each ICB and corresponding ethnic population

**Figure 5:**
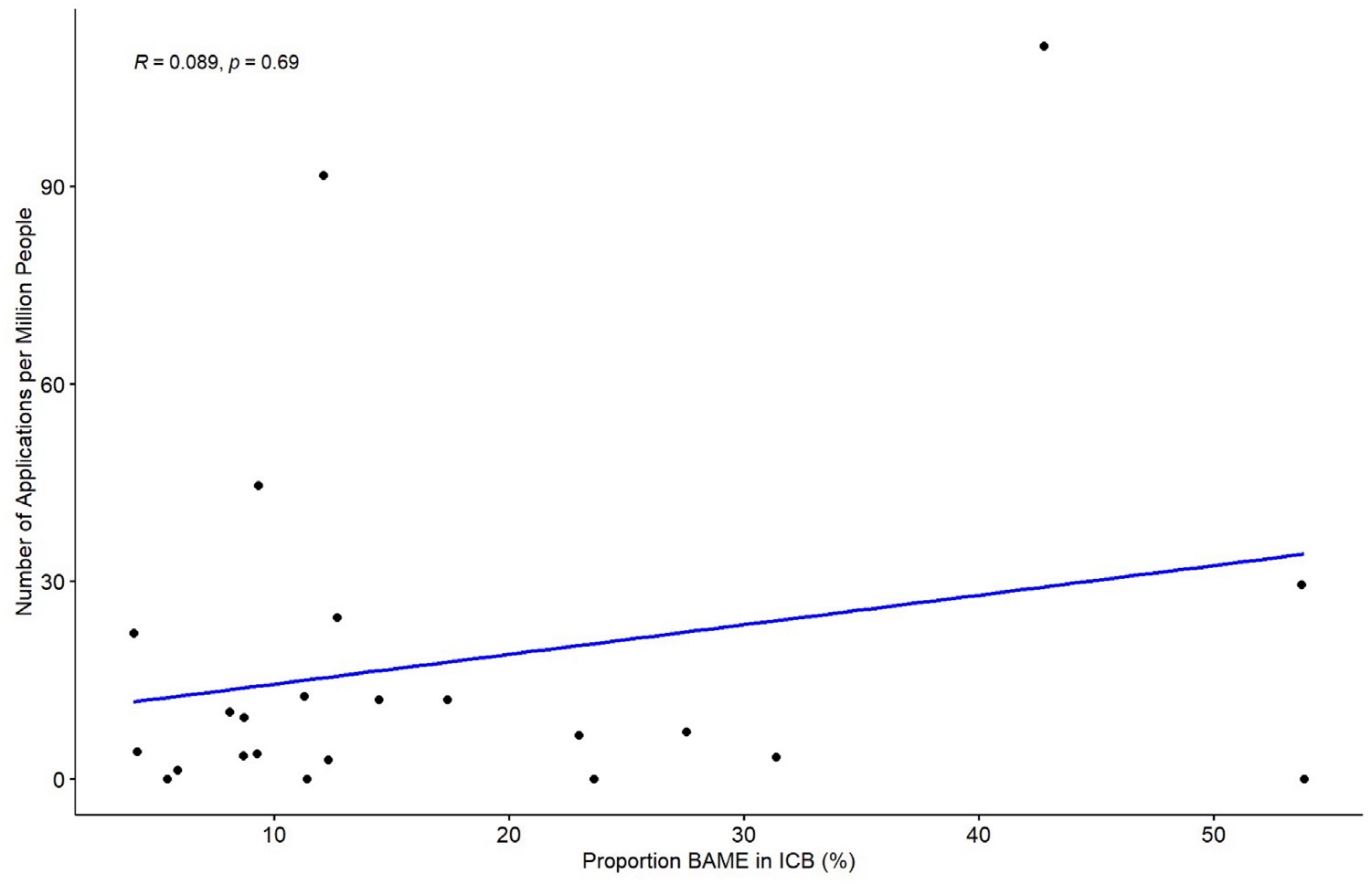
Scatter plot illustrating the relationship between the number of applications per million people and the proportion of BAME individuals within each ICB.

Keloid scar funding applications per million people were compared with the proportion of the relevant ICB’s BAME population (Figure 5). The highest number of applications (111 per million) was made in NHS Central London, which has one of the highest proportions of BAME population (42.8%). However, this correlation was not consistent across all ICBs. A Spearman correlation analysis revealed no statistically significant correlation between the proportion of BAME individuals and number of applications submitted (r = 0.089, p = 0.69). ICBs were omitted from this analysis if the applications per million people had a range of possible values because such ambiguity could skew the Spearman’s analysis. For completeness, the ranges are included in Appendix B.

### Approval of applications in each ICB and THE corresponding ethnic populations

**Figure 6:**
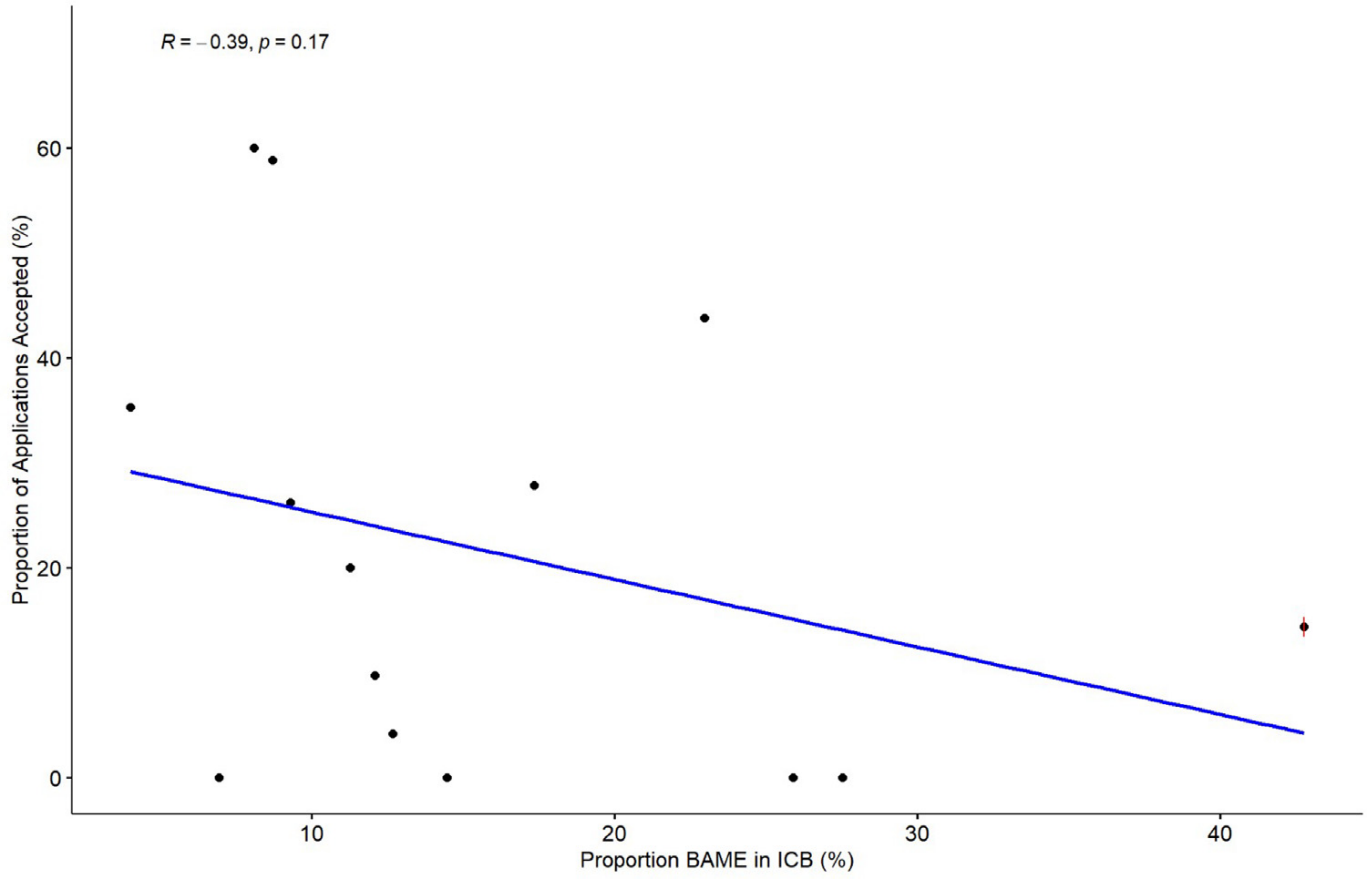
Scatter plot illustrating the relationship between approval rates and the proportion of BAME individuals within each ICB.

The approval rates of funding applications were plotted against the percentage BAME population in each ICB (Figure 6). The Spearman correlation analysis demonstrated no statistically significant association (r = -0.36, p = 0.17).

## Discussion

Health inequalities refer to systematic, unfair and avoidable differences in health outcomes between distinct groups within a population. Identifying and eliminating these disparities should be a focus of health policy. In this context, management of keloid scars warrant scrutiny, given their well-documented association with ethnicity and their potential to engender significant psychosocial distress and unpleasant physical symptoms.^8^^,^^9^

The frequently cited statistic of 15 times higher prevalence in dark-skinned individuals originates from a study by Alhady & Sivanantharajah,^3^ conducted at a single hospital in West Malaysia and it may be an underestimation. More recent studies found higher incidence in West/Central Africa and South India,^7^^,^^10^ with rates ranging from 0.09% in England to as high as 16% in Zaire.^11^ The contributing factors include genetic predisposition, skin pigmentation and cultural practices such as piercing, more prevalent in certain African communities. Logically, restricted access to keloid treatment is likely to disproportionately affect the ethnic minorities.

### why are the current funding Approval Processes considered excessively restrictive?

Our analysis found that keloid surgery is not freely available in any region of England’s NHS based on the assessing clinician’s judgement, with funding restriction applicable in all regions. These funding criteria are variable between regions but are generally highly restrictive.

IFRs and ‘Clinical Exceptionality’

In most regions, surgery is explicitly not permissible without an IFR, precluding a more stream-lined prior approval pathway. Most IFRs apply a principle of ‘clinical exceptionality’ judged by remote gatekeepers without direct knowledge or interaction with the patient.

The key question of clinical exceptionality raises questions of fairness. Based on a two-question test, it is often formulated as the following, or some close variant:

1. Is the patient significantly different from the general population with regard to their condition and at the same stage of progression, **and**
2. Would they be expected to gain significantly more benefits than another patient with the same condition and at the same stage of progression?

In some versions of this test, the clause ‘same stage of progression’ was omitted. The premise of such a test, is that the ‘usual’ presentation of a condition is ineligible for consideration, while only exceptional cases which meet a subjective threshold, are eligible. However, it is not clear, in the context of keloid scarring, how an applicant might satisfy such criteria. This is especially so because severity as a metric appears to be specifically excluded by the clause ‘at the same stage of progression’. Therefore, the IFR process presents an unsolvable riddle, with criteria that are practically impossible to satisfy.

Additionally, the mandated IFR forms are time-consuming for clinicians to complete. They are typically 12 pages in length, necessitating details beyond the clinicians’ remit, such as the costs of treatments, as well as literature reviews of supporting evidence, including analyses of individual studies with respect to clinical effectiveness, exceptionality, resource use and safety. Such a process is labour- and time-intensive for patient-facing clinicians, where the average duration of UK primary care appointments is 9.22 min^12^ and those in secondary care commonly 15 min.

#### Prior approval process criteria

Fundamental to any criteria is the relevance to the condition they purport to assess. Thus, in Dupuytren’s, where the issue is primarily a functional deficit secondary to cords, the criteria relate to the quantitative degree of joint contracture.^13^ In keloids, the widely used criteria often do not assess the true nature of the condition, which seldom leads to functional impairment. Instead, keloids exert distress in other ways, such as psychosocial effect of conspicuous masses and physical symptoms of itch and discomfort.^6^ Such application of inappropriate criteria is to misrepresent the primary nature of a problem, akin to judging Dupuytren’s based on pain or a congenital craniofacial condition solely on function.

In addition to mismatched criteria, subjectivities and technicalities frequently apply. Keloids located on the ears are frequently excluded and those arising from piercings are excluded in most ICBs—explicitly so in 5 and indirectly in several others. This is despite the cultural significance of piercing in ethnic populations and treatment being effective.^14^ The issue is compounded by skin piercing practitioners even in high ethnic areas, being unaware of the risk of keloid formation.^15^ Notably, other lifestyle choices, such as smoking, sun exposure and obesity do not automatically preclude treatment of their sequelae in the NHS.

The National Evidence Based Intervention criteria do not cover keloid scars. Instead, regional ICBs have variously instigated their own criteria, ostensibly without due consideration, given that only 4 of the 40 ICBs recorded ethnicity of the applicants. This significant omission indicates that it is impossible for ICBs to meaningfully audit their policies or perform equality impact assessments. The inception of ICBs in 2022, intended to promote integrated and equitable care, may not be delivering transparency, consistency and public accountability. The salience of this issue is underscored by the evolving demographic landscape of the UK: ethnic minority populations are projected to increase from 13% in 2006 to 28% by 2031, and potentially to 44% by 2056.^16^

### **Factors behind the Low application and acceptance rates**

In an equitable healthcare system, one would anticipate regional parity in the approval rates of treatment applications, alongside a proportional increase in the number of applications in areas with larger BAME populations, given the higher incidence of keloid scarring and its well-documented impact on the quality of life.^3^^,^^4^ However, our analysis found neither scenario to be applicable.

The number of applications did not vary significantly with the proportion of BAME population across the regions. Applications were generally low: 17 out of 34 ICBs reported fewer than 5 applications, with 4 reporting no applications at all (NHS Greater Manchester, NHS Herefordshire and Worcestershire, NHS North West London and NHS Kent and Medway). Although there is limited published data defining the thresholds for anomalously low application rates, an estimated keloid prevalence of 0.09% in England¹¹ equates to approximately 900 affected individuals per million populations. In this context, 5 applications per year represents <0.5% of the potentially eligible individuals pursuing surgical intervention.

There was considerable variation in the application approval rates between ICBs, ranging from 0% to 58.8%, with a mean approval rate of 21.4%. Nottingham and Nottinghamshire ICB (22) were of particular interest. This ICB only records data for benign skin lesions, a category under which they classify keloid scars. Notably, their rate of approving surgery for this category was 71.4%, which far exceeds the reported approval rates specifically for keloids across all other ICBs. This indicates a restrictiveness applied to keloids, which does not appear to apply to other benign skin lesions.

The generally low volume of applications across regions may, in part, reflect the low approval rates observed. Given the significant effort and time investment required by clinicians to make each application, the perceived futility might conceivably result in a deterrent effect. Our snapshot study hints at an underlying feedback loop; historic low acceptance rates act as a negative incentive to apply, possibly explaining the current state of low applications and predicting a further downwards trend.

### Limitations of the study

The study has some limitations. Incomplete responses from FOI requests limits the comprehensiveness of the data. Where values such as ‘<5’ or ‘<10’ were provided, data were interpreted as ranges to avoid unnecessary exclusion. However, these ranges were excluded from the correlation analysis to preserve analytical integrity. Most ICBs did not record ethnicity or Fitzpatrick skin type of the applicants (with ethnicity data provided by only 4 ICBs). This makes it impossible to interrogate any racial disparity in the approval rates of applications. The focus of this study was to explore regional variation in restrictiveness, applications and approval given the already well-established ethnic preponderance in keloid incidence in the literature.

We accept that the ONS’ classification of BAME is a broad category, with a heterogenous composition. Keloid incidence was assumed to be higher in this group compared to White population. However, individual ICB’s BAME populations will vary in ethnic composition, with a resultant unknown variance in the rates of keloid.

Possibly, the rates of keloid are genuinely decreasing, though there is no evidence to support this claim. Being a snapshot study, we could not identify trends in application and acceptance rates over time, which might demonstrate the hypothesised relationship between low acceptance rates and reduction in future applications.

Additionally, given the timeframe of the study, it is important to consider the potential lingering effects of the COVID-19 pandemic as a confounding factor with its deleterious impact on service delivery.^17^ Future studies with a wider scope and longitudinal design might explore these limitations.

## Conclusion

Access to keloid scar surgery is generally highly restricted across England. The process of seeking funding approval is arduous and time-consuming for clinicians, likely accounting for the low number of applications, even in regions serving the highest ethnic minority populations. This, combined with restrictive funding criteria based on inappropriate criteria and low acceptance rates, may plausibly constitute a feedback loop, shaping the clinician’s behaviour, by disincentivising further applications. Health inequality in keloid scar treatment is likely symptomatic of the broader trend towards funding restriction, which curb clinicians’ autonomy, in subservience to remote anonymous gatekeepers. Mandating full equality impact assessments whenever access restrictions are considered could help prevent such health inequalities.

## Ethical approval

Not required.

## Funding

There was no financial support received for this work.

## Declaration of competing interest

The authors declare that they have no conflict of interest.
